# Computed tomography scan radiation doses: Call for optimisation

**DOI:** 10.4103/0971-6203.33239

**Published:** 2007

**Authors:** K. S. Parthasarathy

**Affiliations:** Department of Atomic Energy, Raja Ramanna Fellow, Strategic Planning Group, Board of Research in Nuclear Sciences, Vikram Sarabhai Bhavan, Mumbai - 400 094, India

Preliminary findings from a study released at the annual meeting of the US National Council on Radiation Protection and Measurements held at Arlington, from April 16-18 this year, revealed that doses received from clinical imaging examinations by the US public may have increased by more than 600% in the last two decades; most of it due to computed tomography (CT).[1] The trend is similar in many counties.[2] Regrettably, neither the corresponding estimate nor the statistics regarding CT examinations are available for India.

Another trend in radiological imaging practices that causes greater concern is the increasing number of advertisements appearing in newspapers. Two years ago, a Mumbai (India) daily [The Mumbai Mirror, October 16, 2005] published a colorful advertisement, ‘Know in time,’ with the heading in bold letters, ‘Heart study in 8 seconds’. Another advertisement [The Times of India, December 23, 2005] declared thus: ‘Now discovering the state of your heart vessels is as quick and easy as having a cup of tea.’ The advertisements persuade a potential patient for CT. However, the fact remains that any CT procedure is unjustified if it is not medically necessary.

The first advertisement assured the reader that as a CT procedure was ‘nonsurgical,’ hence, enabling regular check-up of the heart. The advertisement called on patients with ‘stress’ and ‘erratic lifestyle (long working hours, pressure and the like)’ to take the test. As any city dweller may suffer from any one or more such problems, the advertisement aimed at mass screening of asymptomatic persons.

The advertisements did not refer however, to radiation risk. It is thus necessary to disseminate unbiased scientific information about CT among all stakeholders. A few newspapers in India published such articles.[3–7] The risk factors highlighted in the advertisements in the Indian dailies were not identical. Professional associations and leading newspapers could arrest the unbridled use of CT for screening in USA.

The coronary arteries supply blood to heart. They may develop blockages due to formation of plaques, which consist of fat and other substances including calcium. CT can help the physician to get information on the location and extent of calcified plaque formation.

According to *Radiology Info,*[8] a publication of the Radiological Society of North America, not all calcium deposits in the coronary arteries imply blockage and not all blocked arteries contain calcium. Cardiac CT cannot detect soft plaque, the earliest form of coronary artery disease.

The relationship of the calcium score to the likelihood of experiencing angina, myocardial infraction and sudden death remains uncertain. “A screening application is currently not supported by the study data published….I cannot recommend screening application… therefore, aggressive marketing strategies using the method in the context. will face troublesome litigation in most countries,” was the response of Prof. Martin H. K. Hoffmann, Department of Diagnostic Radiology, University Hospital, Ulm, Germany, to an e-mail query.[9]

In 2001, the Atomic Energy Regulatory Board (AERB) of India brought the US Food and Administration's (USFDA) Public Health Notification[10] on reducing radiation risk from CT for pediatric and small adult patients to the then users of over 400 CT units in India. The Board highlighted the need to reduce radiation exposure of children and small adults.

AERB supported a study conducted at the Kerala Health Research Welfare Society Imageology Centre attached to the Medical College, Calicut.[11] It revealed that 12.8% of 5020 consecutive CT examinations were on young patients.

Children are at relatively greater risk as they have more rapidly dividing cells.[10]. According to the US National Research Council's committee on Biological Effects of Ionizing Radiation (BEIR committee), children less than 10 years of age are several times more sensitive to radiation than middle-aged adults.[10] When a CT scan is performed on a child or a small adult with the same technique factors that are used for typically sized adults, the small patient receives a significantly larger dose than the adult. Radiologists or technicians may simply be unaware of the potential dangers from these higher doses. They do not expose the patients knowingly or intentionally.

The FDA notification[10] listed several measures to reduce dose. These include optimizing CT settings, reducing tube current, developing and using a chart or table of tube current settings based on patient weight or diameter and anatomical region of interest, increasing table increment (axial scanning) or pitch (helical scanning), reducing the number of multiple scans with contrast material and eliminating inappropriate referrals for CT. The American Journal Roentgenology (2001) had eight papers on various aspects of CT scan use.[12]

CT scan examinations are immensely useful in diagnosing diseases and trauma and in the guidance of interventional and therapeutic procedures. The individual risk from X-rays associated with a CT scan is quite small compared to the benefits of diagnosis, but it is important to keep the radiation doses during medical X-ray procedures as low as reasonably achievable.

The Atomic Energy (Radiation Protection) Rules 2004 (RPR 2004, www.aerb.gov.in) requires that those who handle CT scan units must have a licence. The radiological safety significance of the practice is thus evident. Unlike Radiation Protection Rules, 1971, the requirements are direct. RPR 2004 clearly specifies the responsibility of the employer, the licensee and the radiation workers. Other countries also have suitable legislation in place. Persistent efforts are needed to ensure regulatory control of this unique tool. We have already lost time in this regard. Regulatory authorities and all other stakeholders must wake up and work harmoniously and promptly to improve radiological safety in CT scan installations.
